# Assessment of Human Visual Acuity Using Visual Evoked Potential: A Review

**DOI:** 10.3390/s20195542

**Published:** 2020-09-28

**Authors:** Xiaowei Zheng, Guanghua Xu, Kai Zhang, Renghao Liang, Wenqiang Yan, Peiyuan Tian, Yaguang Jia, Sicong Zhang, Chenghang Du

**Affiliations:** 1 School of Mechanical Engineering, Xi’an Jiaotong University, Xi’an 710049, China; hlydx1314@stu.xjtu.edu.cn (X.Z.); zhangkai0912@stu.xjtu.edu.cn (K.Z.); lrh8131@stu.xjtu.edu.cn (R.L.); a3115001076@stu.xjtu.edu.cn (W.Y.); tian930724@stu.xjtu.edu.cn (P.T.); jyg.4589815@stu.xjtu.edu.cn (Y.J.); zhsicong@mail.xjtu.edu.cn (S.Z.); d793660193@stu.xjtu.edu.cn (C.D.); 2 State Key Laboratory for Manufacturing Systems Engineering, Xi’an Jiaotong University, Xi’an 710049, China

**Keywords:** visual acuity, visual evoked potential (VEP), stimulus paradigm, threshold determination

## Abstract

Visual evoked potential (VEP) has been used as an alternative method to assess visual acuity objectively, especially in non-verbal infants and adults with low intellectual abilities or malingering. By sweeping the spatial frequency of visual stimuli and recording the corresponding VEP, VEP acuity can be defined by analyzing electroencephalography (EEG) signals. This paper presents a review on the VEP-based visual acuity assessment technique, including a brief overview of the technique, the effects of the parameters of visual stimuli, and signal acquisition and analysis of the VEP acuity test, and a summary of the current clinical applications of the technique. Finally, we discuss the current problems in this research domain and potential future work, which may enable this technique to be used more widely and quickly, deepening the VEP and even electrophysiology research on the detection and diagnosis of visual function.

## 1. Introduction

At least 2.2 billion people around the world are living with vision impairment or blindness, and the risk that more people will suffer from vision impairment can increase due to population growth and aging [1,2,3]. As one of the most important parameters for testing visual ability, visual acuity requires a rapid and precise test method. Visual acuity testing is mainly carried out with subjective psychophysical techniques such as letter charts (Sloan letters or Snellen letters) [4,5] and a partially automated method, the Freiburg Visual Acuity and Contrast Test (FrACT) [6]. However, it is difficult for these subjective methods to assess the visual acuity of examinees with communication problems, such as preverbal children or infants, patients with functional vision problems, those who are mentally disabled, and malingerers [7,8].

Scalp electroencephalography (EEG) provides an alternative method of estimating visual function more objectively and directly, particularly in individuals who experience difficulties in subjective psychophysical testing. Visual evoked potential (VEP) can establish a relationship between visual stimulus and EEG objective response. Hence, since VEP was first used for objective refraction evaluation [9], it has been used to measure visual acuity [10,11,12,13,14,15,16]. By sweeping the spatial frequency of the visual stimulus, e.g., checkerboard or grating, visual acuity can be measured by analyzing the corresponding EEG signals [15].

Many parameters are related to the VEP acuity technique, including the EEG equipment platform (e.g., stimulator, EEG device, etc.), temporal frequency, stimulus field, sweep parameters when the stimulus is presented (e.g., sweep direction, duration, range, etc.), and the visual acuity threshold estimation criterion [17,18]. The validity and reliability of this method and its clinical application have also been widely studied. However, although VEP has been used as an alternative objective visual acuity evaluation method, no common standard guidelines of the technical parameters have been made by researchers.

Hence, we conducted a systematic review of the objective visual acuity evaluation method by the VEP technique. This review aims to gather and synthesize evidence to answer these questions:

(Q1) What is the typical process of the VEP visual acuity assessment technique?

(Q2) What are the widely used and recommended values of relevant parameters (e.g., luminance, contrast, field size, temporal frequency, and electrode placement)?

(Q3) What are the current status and prospects of clinical applications of VEP visual acuity estimation?

The rest of the paper is organized as follows: Section 2 provides an overview of the VEP visual acuity technique. Section 3 discusses the effects of the parameters of visual stimuli on the VEP acuity test. Section 4 discusses the effects of signal acquisition and analysis on the VEP acuity test. Section 5 summarizes the current clinical applications of the VEP acuity technique. Finally, Section 6 discusses the current problems in this research domain and potential future work.

## 2. Overview of VEP Visual Acuity Technique

**Figure 1:**
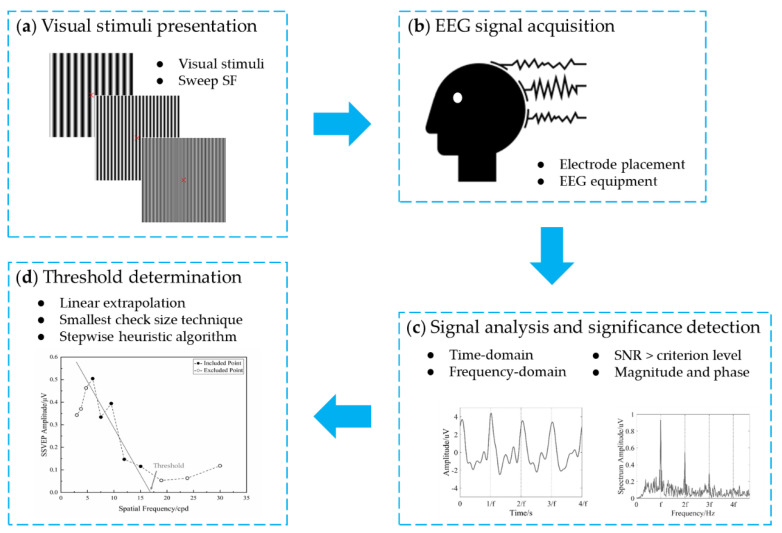
Broad overview of visual acuity assessment by visual evoked potential (VEP). (**a**) Visual stimuli presentation; (**b**) EEG signal acquisition; (**c**) signal analysis and significance detection; and (**d**) threshold determination. SF, spatial frequency.

Figure 1 shows an overview of the VEP visual acuity technique, mainly containing four steps: visual stimuli presentation, EEG signal acquisition, signal processing and analysis, and VEP acuity threshold determination. First, visual stimuli are presented to the examinee on a display with the spatial frequency sweeping over time. Then, the EEG acquisition device records the VEP signals from electrodes on the occipital area, e.g., Oz. Next, a signal analysis algorithm, e.g., discrete Fourier transform (DFT), is used to analyze the VEP signals. The significance of the VEP response for each spatial frequency is objectively detected by the corresponding criterion, e.g., signal-to-noise ratio (SNR). Finally, the threshold determination algorithm is used to define the VEP visual acuity threshold generally by extrapolating VEP magnitude versus spatial frequency to some baseline, or an alternative technique of the finest spatial frequency evoking a significant VEP.

## 3. Effects of Visual Stimuli

### 3.1. Luminance

**Figure 2:**
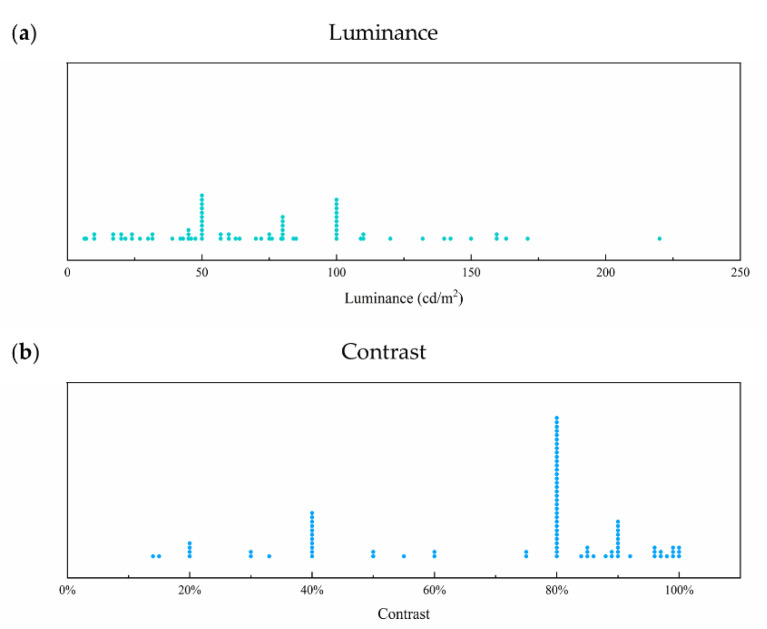
(**a**) Luminance and (**b**) contrast of visual stimulus in selected studies. Each point represents one luminance or contrast value used in one of the studies.

As shown in Figure 2a, most studies used different levels of luminance ranging from 6.36 to 220 cd/m^2^ (mean ± SD: 71.99 ± 43.26 cd/m^2^), and the most used luminance was concentrated on 50 and 100 cd/m^2^. Allen et al. [19] illustrated that the VEP acuity of infants and adults increased with luminance increasing from 0.01 to 1 cd/m^2^ and remained asymptotic between 1 and 100 cd/m^2^. Good et al. [20] found that children with cortical visual impairment (CVI) had more outstanding VEP acuity when the stimulus showed a low-luminance background, while there was no difference in VEP acuity between low- and high-luminance backgrounds in children without such impairment. In addition, when presenting the visual stimulus, the mean luminance of the stimulus should be the same as the background luminance, since changes in luminance may evoke VEP in individuals who cannot resolve the pattern stimulation [21].

### 3.2. Contrast

The contrast of stimulus, Michelson contrast, is defined as follows:

(1) $$\mathrm{Contrast}\;=\;\frac{L_{w}-L_{b}}{L_{w}+L_{b}}\times 100\%$$

where *L_w_* and *L_b_* are the luminance of the white and black checks or stripes in the visual stimulus of the checkerboard or grating, respectively.

As shown in Figure 2b, the contrast of the stimulus ranged from 14% to 100% (mean ± SD: 71.97% ± 23.71%), and the most used contrast was concentrated on 80%. However, Bach et al. [22] suggested using a slightly lower contrast of 40% for the following reasons. First, a moderate contrast was enough to evoke full amplitude, since the contrast transfer function of VEP was saturated in advance [23]. Second, the gamma-correction of the screen could be easily accomplished when the luminance value was not so extreme with the rather low contrast [24,25]. Third, luminance artifacts, a danger in onset stimulus, can be more easily avoided. The medium contrast of 40% was also used in their other studies [26,27,28,29,30,31].

### 3.3. Stimulus Pattern

**Figure 3:**
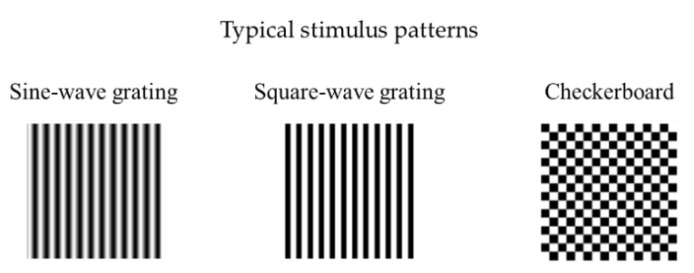
Three typical stimulus patterns of sine-wave and square-wave gratings, and checkerboards used in VEP acuity assessment.

**Figure 4:**
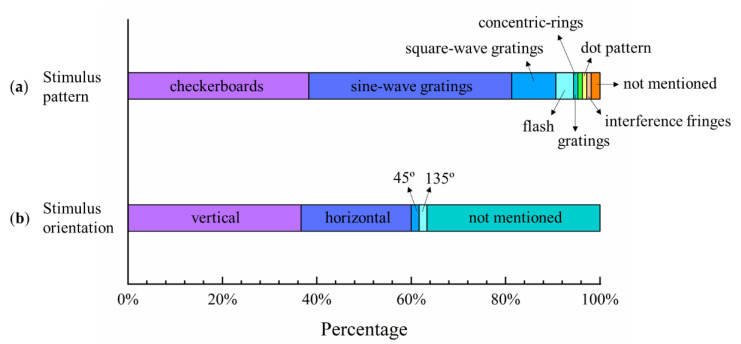
Percentages of (**a**) stimulus patterns and (**b**) grating orientations used in selected studies of VEP acuity assessment.

Several stimulus patterns were used in the VEP acuity assessment, and Figure 3 shows three typical patterns used widely in the relevant studies: sine-wave gratings, square-wave gratings, and checkerboards. The gap between the neighboring black and white parts represents the spatial frequency of the stimulus. Some studies used a simple pattern of flashes to predict the final visual acuity of patients with cataracts, diabetes, and other severe ocular injuries by VEP [32,33,34,35]. Towle and Harter [10] used a dot pattern rather than a checkerboard since the perception of the dot pattern would be less influenced by cylindrical refractive errors. Simon and Rassow [36] used a laser interferometer to generate interference fringes as the stimulus pattern. Zheng et al. [7] designed a concentric-ring pattern with oscillating expansion and contraction to arouse steady-state motion visual evoked potential (SSMVEP), which had the properties of low sensitivity to changing contrast, low mental load, and less visual fatigue [37,38,39]. Tobimatsu et al. [40] recorded transient and steady-state VEP to the reversal of checkerboard, square-wave grating, and sine-wave grating patterns to assess the contributions of the fundamental spatial frequency and higher harmonic segments, and recommended that the fundamental spatial frequency of the pattern affected the VEP response. Figure 4a shows the percentages of studies that used different stimulus patterns. Checkerboard and sine-wave grating were the most used patterns, with proportions of 39% and 44%, respectively. The gratings in Figure 4a refer to unknown descriptions of gratings used with either sine-waves or square-waves in a previous study [41].

### 3.4. Stimulus Orientation of Grating Pattern

The orientation of the visual pattern is another parameter that affects the performance of VEP visual acuity assessment, since the orientation effect has been shown for several psychophysical visual tasks, such as grating acuity and contrast sensitivity [42,43,44]. Previous studies proved that VEP was affected by stimulus orientation, and the VEP response was lower to obliquely oriented gratings than to vertically oriented gratings [45,46,47]. Arakawa et al. [48] investigated the impact of stimulus orientation on the VEP response over various spatial frequencies of sine-wave gratings at four orientations (vertical, horizontal, and oblique at 45° and 135°), and found that there were different VEP spatial frequency functions between oblique and vertical/horizontal gratings. Figure 4b shows the percentages of studies that used the different stimulus orientations of gratings; vertical and horizontal orientations were the most used visual orientations, with proportions of 37% and 23%, respectively.

### 3.5. Stimulus Mode

**Figure 5:**
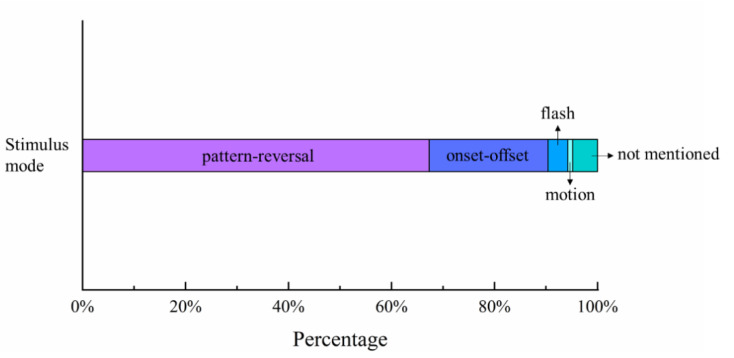
Percentages of stimulus modes used in studies of VEP acuity assessment.

Figure 5 shows the percentages of stimulus modulated modes in the selected studies. Except for the motion mode of oscillating expansion and contraction [7] and the flash mode in several studies [32,33,34,35], the most used stimulus modes were pattern-reversal and pattern onset-offset, with proportions of 71% and 24%, respectively.

**Figure 6:**
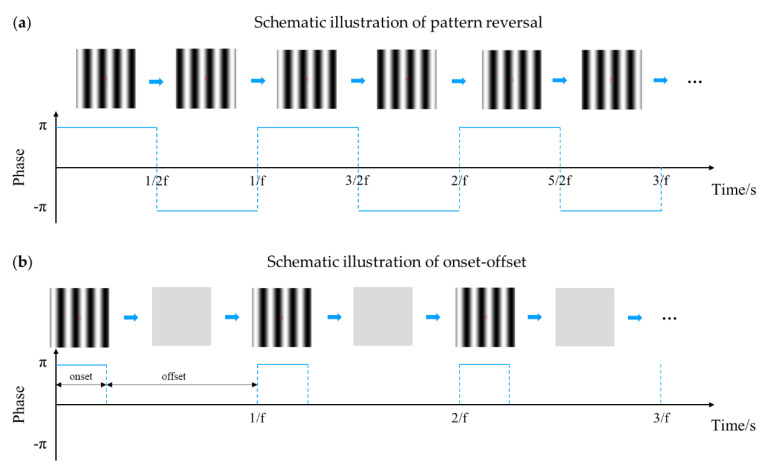
Schematic illustration of pattern-reversal and onset-offset stimulus modes of vertical sine-wave gratings. f, fundamental temporal frequency. (**a**) Schematic illustration of pattern reversal. (**b**) Schematic illustration of onset-offset.

The two common modes of pattern-reversal and onset-offset are illustrated schematically in Figure 6, taking vertical sine-wave gratings as an example. In the pattern-reversal mode, a grating alternates between two states in which the bright parts shift to the dark parts of an equivalent luminance value and vice versa, with the mean luminance of the whole pattern remaining constant. The EEG spectrum contains only even harmonics in the frequency-domain of steady-state visual evoked potential (SSVEP) [16,49,50]. The onset-offset mode presents a grating pattern alternating with a uniform gray field with the same mean luminance. The visual system was shown to have a large response after the pattern transited from the gray field to the grating pattern, i.e., the onset, but a small one at the offset; the resulting response was presented at the fundamental temporal frequency and possibly higher harmonics in SSVEP [16,51].

### 3.6. Stimulus Field

**Figure 7:**
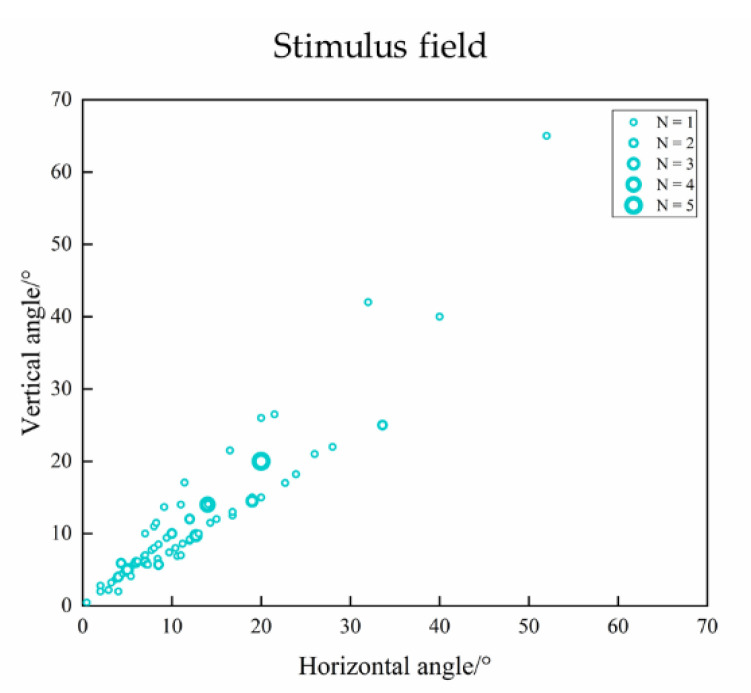
Visual angle of stimulus field in selected studies of VEP acuity assessment.

As shown in Figure 7, the visual angle of the stimulus field ranges from 0.465 × 0.465° to 52 × 65° (horizontal × vertical, mean ± SD: 12.77° ± 9.08° × 12.16° ± 9.86°). In some studies, the testing distance was determined based on the patient’s age and visual behavior, so the visual angle would change accordingly [52,53,54,55]. Finer patterns may evoke foveal VEPs, while coarser patterns may also evoke extrafoveal VEPs [56]. Hence, the VEP amplitude can be affected by the stimulus field size, especially in low spatial frequency, and the amplitude decreases with decreasing stimulus area [57,58]. Tyler et al. [59] used an annular stimulus ranging from 2° to 15° in inner diameter with a constant outer diameter and found that the estimated visual acuity did not change with the changed stimulus area, although the VEP amplitude decreased at a low spatial frequency as the stimulus area decreased. Almoqbel et al. [17] used two pieces of white cardboard to mask the stimulus screen and form a stimulus field of 6, 4, or 2° and found that the stimulus area affected visual acuity, suggesting a stimulus area of 4° or larger for VEP acuity assessment. The effect of the stimulus field on the VEP amplitude may be caused by the number of neurons induced by the stimulus in the visual field, so a larger stimulus area could evoke a wider cortical response, resulting in greater VEP amplitude.

### 3.7. Temporal Frequency

**Figure 8:**
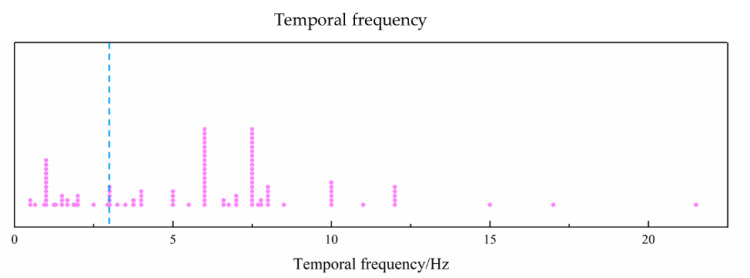
Temporal frequency of visual stimulus in selected studies. Dashed blue line represents critical point of 3 Hz distinguishing transient and steady-state VEP.

For consistency, the temporal frequency of the stimulus of pattern-reversal mode is defined as the frequency at which the stimulus returns to its original state [16]. Hence, for example, the temporal frequency of two reversals per second (rps) is 1 Hz in pattern reversal mode, which was also defined in many studies [60,61,62,63]. We took the uniform unit of Hz in all stimulus modes for statistical analysis. Figure 8 shows the temporal frequency used in the selected studies; the dashed blue line represents the critical point of 3 Hz distinguishing the transient and steady-state VEP [40,64]. The most used frequencies were 1 Hz for transient VEP, and 6 Hz and 7.5 Hz for SSVEP. We can conclude that most studies used the SSVEP technique combined with a frequency-domain analysis method for EEG data, such as Fourier transform, obtaining the amplitude and phase at a specific frequency, e.g., the fundamental frequency of onset-offset mode or second harmonic of pattern reversal mode [27,56,61,65,66]. On the other hand, the transient VEP with a temporal frequency lower than 3 Hz used a time-domain analysis method such as a superimposed average, obtaining the amplitude and latency of a time-domain peak, e.g., P100 [67,68,69,70,71,72].

As for the temporal frequency of visual stimulus in the studies of VEP visual acuity assessment, Regan [56] compared the temporal dependence of spatial frequency selectivity and found that VEP to a stimulus of high spatial frequency was largest at a low temporal frequency (e.g., 5–7 Hz), whereas stimulus of low spatial frequency gave the largest VEP at a high temporal frequency (e.g., 10 or 17 Hz). Norcia and Tyler [13] found that there was no significant difference in infant VEP acuity at temporal frequencies of 6 and 10 Hz. Sokol et al. [73,74] found that grating acuity was temporally tuned in infants, but not in adults. The acuity was higher at a medium temporal frequency (e.g., 7.5 or 14 rps) than at a low or high temporal frequency (e.g., 2.5 or 23 rps) in infants. The difference between psychophysical acuity, e.g., preferential looking (PL) acuity, and VEP acuity may change with various temporal frequencies of the visual stimulus, converging to nearly equivalent levels by 12 months of age. Gottlob et al. [75] compared the correlation between subjective and VEP acuity at four stimulus frequencies (8, 12, 15, and 24 rps), and found high correlation coefficients for each temporal frequency. Almoqbel et al. [17] measured sweep VEP (sVEP) visual acuity at three temporal frequencies (6, 7.5, and 10 Hz) and found that there was more viable visual acuity in both children and adults at 7.5 Hz than at 10 or 6 Hz, and suggested 7.5 Hz as the temporal frequency in VEP acuity tests.

### 3.8. Sweep Parameters

Sweep VEP (sVEP) is measured in response to a visual stimulus that is parametrically swept within a range of values instead of a fixed and constant value. Sweep VEP is usually used in objective tests for visual acuity assessment by sweeping the spatial frequency over time [17]. Some sweep parameters may impact the threshold obtained with sVEP, such as type (logarithmic vs. linear sweep), direction (low-to-high vs. high-to-low spatial frequency), range of spatial frequency, mode (continuous or stepwise), and duration.

#### 3.8.1. Sweep Mode

As for the sweep mode [76], when sweeping continuously, the spatial frequency of a stimulus paradigm, e.g., grating, changes continuously during one sweep [74,77]. The VEP for a certain spatial frequency is extracted by a narrow-band synchronous filter technique [59,78]. Similarly, a stepwise sweep consists of a series of gratings with different spatial frequencies. A given spatial frequency is presented for a fixed period time, e.g., 0.5 s [19,75,79], 1 s [80,81], or 2 s [82], and then the stimulus is changed to the next spatial frequency [83,84]. The VEP data processing method, e.g., DFT, is obtained from the fixed period for the corresponding spatial frequency [85,86]. Most of the relevant studies used stepwise sweeps [15].

#### 3.8.2. Sweep Direction

Sweep direction is another sweep parameter that may affect VEP acuity. The most used direction is low-to-high spatial frequency [7,81]. Almoqbel et al. [17] compared the two sweep directions of spatial frequency and did not find a significant difference. Hemptinne et al. [87] used two sweep directions of gradually increasing and decreasing spatial frequency of the gratings to measure SSVEP visual acuity, and found that the correlation between increasing and decreasing spatial frequency was quite high.

#### 3.8.3. Sweep Type

Another parameter to be considered is the sweep type, i.e., linear or logarithmic sweep: the spatial frequency is presented as a linear or logarithmic increase or decrease [15]. Tyler et al. [59] used a linear instead of a logarithmic method of sweeping spatial frequency to minimize the delay in the visual response due to the time constants of the synchronous filter and the brain response and found that the linear sweep technique was highly accurate. Gottlob et al. [75] used logarithmic sweep to make sure the anticipated acuity was within the upper third of the sweep range when the sweep range of spatial frequency was uncertain. Zhou et al. [61] and Kurtenbach et al. [18] recommended logarithmic instead of linear sweep and found that the estimated VEP acuity from linear extrapolation from the VEP amplitude peak to 0 µV baseline against log visual-angle/log spatial frequency was not significantly different from subjective visual acuity. Bach et al. [22] used a stepwise heuristic algorithm to sweep spatial frequency in logarithmic steps to determine the optimal range for the regression line. The VEP acuity obtained from normal, artificially reduced, and reduced acuity with the ocular disease was highly related to behavioral acuity. Recently, Hoffmann et al. [30] used a logarithmically equidistant step of spatial frequency from 0.52° to 8.9° to explore whether VEP could be extended for low-vision evaluation and found a good agreement between psychophysical and electrophysiological visual acuity in the low-vision range down to 2.0 logMAR. Hemptinne et al. [87] also used a logarithmically spaced value to sweep the spatial frequency from 2.7 to 40 cpd. In total, if the anticipated acuity is uncertain or the sweep range is slightly large, a logarithmic sweep can make the spatial frequency of the stimulus approach the visual limit rapidly. Otherwise, a linear sweep may be more accurate and detailed. In fact, a linear sweep was applied more in previous studies [29,46,88].

#### 3.8.4. Sweep Duration

Sweep duration is the time required for one sweep between the lowest and highest spatial frequency. The main advantage of sVEP, which is widely used in VEP acuity assessment, is its shorter recording time than conventional VEP [17,89]. In general, the sweep duration ranges from 10 s to 20 s [13,54,59,62,65,74,80,82,85,87,90,91,92,93,94]. Ridder et al. [95] found that the change of the amplitude of the VEP with continued stimulus presentation did not affect the sweep VEP acuity estimate, indicating a minimum for sweep VEP duration (1–2 s per spatial frequency) to optimize the total test time. Almoqbel et al. [17] used three sweep durations of 10.7, 14.7, and 20 s, finding that sweep durations between 10 and 20 s did not affect the extrapolated threshold. Iyer et al. [96] introduced an SNR measurement, Fps, to reduce the sweep time by assessing the signal quality in order to minimize the amount of sweep data required for VEP acuity evaluation.

#### 3.8.5. Sweep Range

**Figure 9:**
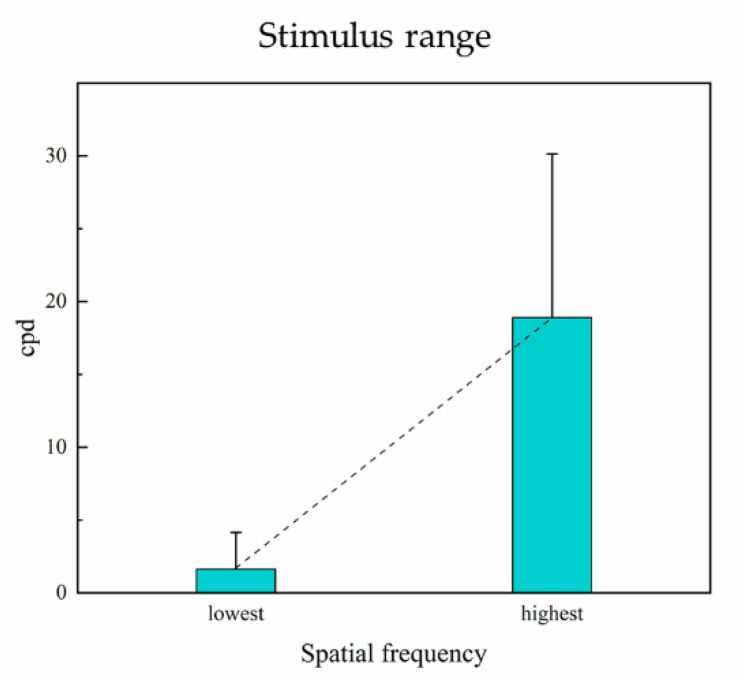
Mean sweep range of lowest and highest spatial frequencies in selected studies. Error bars: SD.

Sweep range is also an important parameter in the VEP acuity test. To obtain better VEP results, preferably, most of the sweep spatial frequencies should be recognizable with a significant SNR to extrapolate the amplitude against spatial frequency. The sweep range of spatial frequency is about 3 to 30 cpd, corresponding to psychophysical optotypes from 1.0 to 0.0 logMAR in normal humans [7,36,77,83,85,87,97,98]. Besides, the sweep range was biased toward lower spatial frequencies in studies on infants and depended on their age, since their visual function develops over time [13,19,20,52,65,66,81,88,99,100,101]. For patients with visual disorders or low vision, the sweep range was also lower than the normal value [12,20,22,26,30,52,88,102,103,104,105,106,107]. The mean sweep range in selected studies is shown in Figure 9.

In summary, the luminance of visual stimuli for the VEP acuity technique mainly ranges from 50 to 100 cd/m^2^. Higher contrast can achieve a better SNR but may enhance the risk of amplitude notch at intermediate spatial frequencies. Lower contrast, e.g., 40%, can avoid luminance artefacts and be more comfortable to view. Sine-wave gratings, square-wave gratings, and checkerboards are widely used. The orientation of gratings, e.g., horizontal vs. vertical, does not affect the VEP acuity, but oblique orientations have poorer VEP acuity. Pattern-reversal and onset-offset modes are mainly used, but care is needed concerning the problem of luminance artefacts in onset-offset mode. A stimulus field ranging from around 4 to 10° has no effect on VEP acuity, and a stimulus area of 4° or larger is suitable for assessment. A temporal frequency of 10–24 rps for pattern-reversal or 5–12 Hz for onset-offset is widely used. As for sweep parameters, stepwise sweeps are commonly used. The sweep direction does not affect VEP acuity, and the direction of low-to-high spatial frequency is mostly used. Compared to logarithmic sweep, linear sweep may be more accurate when spatial frequency is close to the threshold, but logarithmic sweep can make the spatial frequency of the stimulus approach the visual limit rapidly. Sweep duration mainly ranging from 10 to 20 s has no effect on VEP acuity. The range of spatial frequency should include sufficient data points to approach or bracket the VEP SF limit, and in general, an upper limit of 30 or even 40 cpd is needed.

## 4. Effects of Signal Acquisition and Analysis

### 4.1. VEP Acuity Assessment System

The VEP system mainly contains hardware (e.g., stimulus screen, data acquisition device, and input amplifier) and software (e.g., stimulus generator, EEG data processing program, and visual acuity threshold algorithm). There are two types of VEP acuity assessment systems. One combines the independent parts into a system, called a combined system, such as the systems in previous studies [7,35,36,68,75,104]. The other is a dedicated system utilized to generate the visual stimulus, record the VEP, and define the acuity in VEP assessment with some necessary auxiliary equipment, such as the Enfant system (Neuroscientific Corp., Farmingdale, NY, USA) [61,62,63,80,82,85,93,94,95,108], the PowerDiva system developed by Norcia (Smith-Kettlewell Eye Research Institute, San Francisco, CA, USA) [13,14,17,52,75,79,83,99,109,110,111], the RETIsystem (Roland Consult Instrument GmBH, Wiesbaden, Germany) [69,71,72,77,112], and the Freiburg Evoked Potentials (EP2000) system developed by Bach (Medical Center, Freiburg University, Freiburg, Germany) [22,30,31,113]. Bach and Farmer [26] evaluated the implementation and outcome quality of the VEP acuity method on commercial equipment (Diagnosys Espion Profile and E3 electrophysiology systems) and found that VEP acuity agreed with subjective acuity to within ± 0.31 logMAR, indicating that this technique can be accessible to more users. In addition, Ridder et al. [97] compared the two commonly used systems, Enfant and PowerDiva, demonstrating that the acuity estimates with these systems did not have significant differences for normal subjects.

### 4.2. Electrode Placement

**Figure 10:**
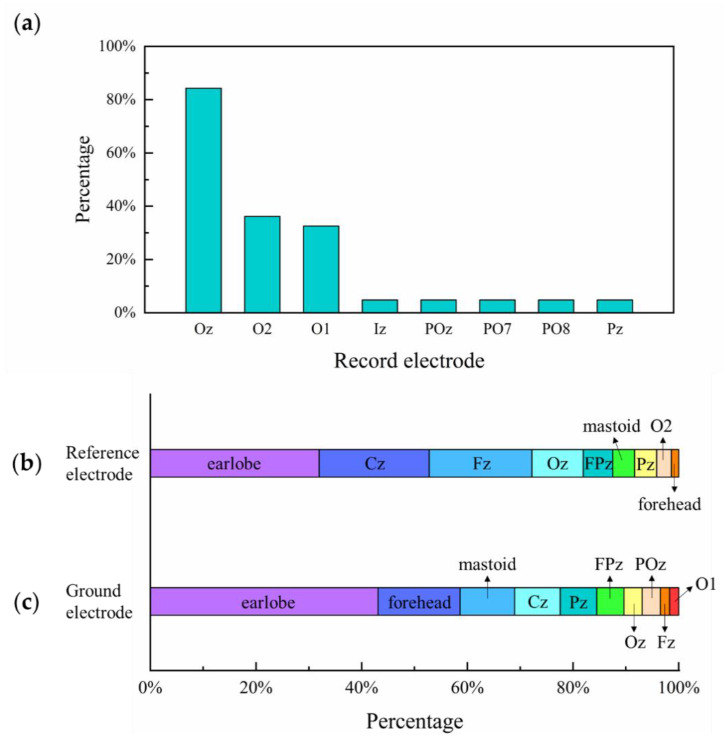
Electrode placement and percentage of (**a**) recording, (**b**) reference, and (**c**) ground electrodes in selected studies.

VEP to visual stimulus of different spatial frequencies was traditionally recorded at the middle occipital area, i.e., electrode Oz [87], which was put above the inion at 10% of the measured distance between the inion and the nasion over the vertex, according to the International standard 10–20 system and International Society for Clinical Electrophysiology and Vision (ISCEV) standard electrode locations [21,114]. According to the standard head sizes of infants, children, adolescents, and adults [115,116], we converted some descriptions of recording electrode placement using relative position rather than electrode name (e.g., a bipolar derivation of 1 cm over the inion versus 3 cm to the right at the same level [13,14]) into standard electrode position. As shown in Figure 10, we concluded that over 80% of previous VEP acuity studies used Oz as the recording electrode, with the reference electrode mostly placed at Fz [21], Cz, earlobe, and Fpz. The commonly used ground electrode positions included the forehead, vertex (Cz), mastoid, and earlobe (A1 or A2) [21].

Among these studies, Almoqbel et al. [17] found that there was no significant effect between ISCEV and PowerDiva electrode placements. Hemptinne et al. [87] tested sVEP acuity in adults with a 68-electrode EEG system (64-channel Biosemi Active 2 system with four extra electrodes, PO9, PO10, I1, and I2, over the occipitotemporal area) and recommended the most sensitive electrodes (Iz, Oz, POz, O1, PO7, O2, and PO8) in VEP acuity assessment. Most studies used only one recording electrode on the occiput to collect EEG signals, and some studies used more than one electrode, e.g., three recording electrodes of Oz, O1, and O2 [26,27,117]. To improve the SNR of VEP, the spatial filtering method, which linearly fuses multi-lead signals into single-channel signals [118,119], was used to fuse multi-channel VEP signals. For example, a Laplacian transform was used in some studies with three recording electrodes [18,22,26,27,28,30,31,89,105,120], and canonical correlation analysis (CCA) fusion was used in our previous study [7].

### 4.3. Threshold Determination Method

The threshold determination method was the VEP acuity threshold estimation by VEP response against spatial frequency. We classified the threshold determination methods mentioned in selected studies, as shown in Table 1. The most widely used method was the linear extrapolation from the highest VEP amplitude response to the 0 µV amplitude baseline between VEP response amplitude and spatial frequency, and this spatial frequency of intersection with the *x*-axis (zero-response amplitude baseline) was determined as the VEP estimate of acuity. This linear extrapolation method was first proposed by Tyler et al. [59] and subsequently used in many studies [13,14,17,19,20,36,62,63,65,66,73,74,75,79,80,81,82,85,86,88,90,91,92,93,94,95,96,98,99,100,101,102,103,106,108,110,121,122,123,124,125,126,127,128,129,130], and even in recent studies [52,77,97,109,117]. If there are multiple VEP amplitude peaks, the last peak with the highest spatial frequency should be selected to extrapolate the threshold [13,14]. Moreover, the extrapolation technique takes into account the SNR and phase statistics. The SNR of an exact value, e.g., peak SNR ≥ 3:1 [14,17], was used as a criterion for accepting a given visual acuity threshold. The phase of the VEP response was constant or gradually lagged the stimulus with increasing spatial frequency since the latency increased with increased spatial frequency [14,15,17].

Some studies tried some optimizations of the linear extrapolation method. Except extrapolating to zero amplitude, visual acuity was also reckoned as the spatial frequency of intersection by extrapolating from the VEP amplitude peak to the noise level [46,80,83,121,126,127,129], which was not significantly different from 0 µV extrapolation [80]. Zhou et al. [61] estimated VEP acuity using both VEP amplitude–spatial frequency and VEP amplitude–log visual-angle function and found that the VEP amplitude–log visual-angle function regression method was more accurate in normal subjects. Kurtenbach et al. [18] plotted VEP amplitudes corresponding to fundamental frequency against log spatial frequency, and used the regression line fitting the descending trend of the VEP amplitude response function and extrapolating to zero, and then took 23 cpd equal to the acuity of 0.0 logMAR.

Another threshold determination method, which is more direct and faster, is called the smallest check size technique [10,53,67,84], taking the smallest check size that evokes a repeatable and recognizable VEP as the VEP acuity [131], which was also used in other studies [71,72,105,111,132]. To improve the smallest check size technique, Mackay et al. [89] used a successive approximation algorithm to generate the stimulus, and the visual acuity threshold was defined when the VEP response to three consecutively increasing spatial frequencies was scored as detection, detection, no detection. Hemptinne et al. [87] defined the visual acuity threshold as the epoch preceding the last epoch with a significant VEP response and having a significant VEP response in at least three of the four previous steps, which may be able to resolve some mistakes of VEP response at supra-threshold spatial frequency. Similarly, Zheng et al. [7] combined the significance of the VEP response and the “OR” algorithm in Boolean algebra to solve the problem that VEP response can be influenced by the external environment and mental state of subjects, especially when the parameters correspond to the tiny super-threshold.

Bach et al. [22] proposed a stepwise heuristic algorithm that was used in later studies [26,28,31,120], which avoided the “notch” at intermediate check sizes [133,134,135,136]. This method can find an optimal range for the regression of noise-corrected VEP amplitude vs. log spatial frequency, yielding a value of SF_0_, the log spatial frequency limit where the VEP amplitude is extrapolated to zero, via a set of rules on VEP amplitude and noise estimates. Finally, Freiburg VEP acuity, with a dimension of one over degrees, can be obtained by dividing SF_0_ by 17.6 [22].

As for other threshold determination methods, Jenkins et al. [55] used the extrapolation of the curvilinear function of the best-fitting quadratic equation to zero amplitude, with the check size of the extrapolated intercept as the threshold. Kurtenbach et al. [18] plotted the peak amplitude against spatial frequency and fitted the data with a second-order polynomial function and a manually set cursor and obtained the limiting spatial frequency from the intercept of the *x*-axis, and then took 23 cpd to be equal to the visual acuity of 0.0 logMAR. Strasser et al. [137] used multiple linear regression, i.e., a second-order polynomial, and nonlinear regression, i.e., a modified Ricker model, to fit VEP peak amplitudes and spatial frequency, and found that the two models performed equally well in predicting visual acuity. Moreover, the modified Ricker model was more reliable and robust than the second-order polynomial model, since it did not require the exclusion of data points from the fit [137]. Furthermore, recently, Bach and Heinrich [27] used a machine learning approach with a small dataset of 108 cases [22] to transform VEP results into visual acuity automatically. They tested more than 100 algorithms and found that rule-based and multiple regression methods performed best. They concluded that the machine learning approach appeared to be an alternative method that was useful for the analysis of acuity VEP data [27].

In summary, the widely used systems in VEP acuity estimation are dedicated systems or custom designs that are not sold commercially or have official approval, restricting the promotion and application of this technology. Active electrodes close to Oz are used to record VEP signals sometimes with spatial filtering methods, especially Laplacian montage, to enhance SNR. VEP acuity is usually defined by linear extrapolation of significant VEP magnitudes versus spatial frequency. The intercept of 0 µV is often defined as the VEP acuity threshold. When the extrapolation technique fails to define a VEP acuity threshold, mainly because the VEP magnitudes are poor due to the deep notch at an intermediate spatial frequency, an alternative method, the smallest check size technique, can be used to define VEP acuity.

**Table 1:** Threshold determination methods in VEP acuity test.

Threshold Determination Method	Description	Studies
Linear extrapolation	Linear extrapolation from last VEP amplitude peak to 0 µV baseline versus linear spatial frequency	[13,14,17,19,20,36,52,59,62,63,65,66,73,74,75,77,79,80,81,82,85,86,88,90,91,92,93,94,95,96,97,98,99,100,101,102,103,106,108,109,110,117,121,122,123,124,125,126,127,128,129,130]
Improved linear extrapolation	Linear extrapolation from last VEP amplitude peak to 0 µV baseline against log visual–angle/log spatial frequency	[18,61]
	Linear extrapolation from last VEP amplitude peak to noise level baseline against spatial frequency	[46,80,83,121,126,127,129]
Smallest check size technique	Smallest check size that evokes a recognizable and repeatable VEP	[10,53,67,71,72,84,105,111,132]
Improved smallest check size technique	Three consecutively increasing spatial frequencies: detection, detection, no detection	[89]
	Significant response among at least three of the four preceding steps	[87]
	Significance of VEP response combined with OR algorithm in Boolean algebra	[7]
Stepwise heuristic algorithm	Optimal range for regression and value for SF_0_ or failure for all VEP recordings via a set of rules on VEP amplitude and noise estimate	[22,26,28,31,120]
Other methods	Extrapolation of curvilinear function of best-fitting quadratic equation to zero amplitude	[55]
	Second-order polynomial function plotting peak amplitudes against spatial frequency	[18,137]
	Nonlinear regression of modified Ricker model fitting sweep VEP peak amplitudes and spatial frequency	[137]
	Machine learning approach with small dataset of 108 cases	[27]

## 5. Clinical Application

### 5.1. Studies of the Visual Acuity Development by VEP

VEP has also been used in research on visual acuity development, especially in infants [15], as summarized in Table 2. Some studies gave acuity improvement values with increased age. For example, acuity increased from 20/150 at two months to 20/20 by six months [100], from 4.5 cpd at the first month to about 20 cpd at 8–13 months [14], from 6 cpd at 2–10 weeks to 14 cpd at 20–30 weeks [124], from 2.5–9 cpd during the first two months to about 10–20 cpd after 30 weeks [65], from 9.61 cpd at 4 months to 10.39 cpd at eight months [93], and from 0.80 logMAR in the first month of life to 0.06 logMAR at 36 months of age [99], and acuity improved by a factor of 2.3 between 10 and 100 weeks [66].

In addition, some studies compared VEP acuity with subjective acuity, e.g., TAC acuity [138], PL acuity, or forced-choice preferential-looking (FPL) acuity [139]. For example, Sokol et al. [73,74] found that the difference of VEP and PL acuity decreased from 2.0 octaves at two months to 0.5 octaves at 12 months, and VEP and PL acuity developed at different rates, converging to nearly equivalent levels by 12 months. Allen et al. [19] found that VEP and FPL acuity were comparable, with VEP acuity slightly higher. Riddell et al. [121] tested VEP and TAC acuity on infants and found that VEP acuity was generally higher than TAC acuity, but the rate of TAC acuity development was steeper, with the two converging to the same level at about 14 months. Almoqbel et al. [109] compared the results of various procedures (sweep VEP, psychophysical logMAR letter, and grating visual acuity) on children and found no difference.

Several studies also verified the performance of VEP acuity assessment in infants [35,81]. Moreover, other studies analyzed the effect parameters for assessing visual acuity development, such as temporal frequency [13], front-end nonlinear distortion products [91], and perinatal characteristics including birth weight, gender, and the number of smokers in the household [101].

### 5.2. Clinical Studies of VEP Acuity Assessment

The VEP technique was identified as a valuable and alternative approach for evaluating visual acuity not only in normal vision but also in visual disorders [15]. As shown in Table 3, we summarized the previous studies that used VEP as the visual acuity evaluation method for disorders that may affect vision, e.g., cortical/cerebral visual impairment (CVI), amblyopia, cataract, hydrocephalus, glaucoma, albinism, nystagmus, etc.

For patients with CVI, Good [90] used sVEP as a quantitative tool to measure vision, since youngsters with CVI tend to stare at lights, i.e., visual stimulus, indicating that the sVEP approach is a reliable and valid tool for evaluating visual acuity in children with CVI. Good and Hou [20] illustrated the sVEP acuity of children with CVI in two luminance conditions (normal: 109 cd/m^2^; low: 20 cd/m^2^), showing that their visual acuity was better at low luminance than at normal luminance, which was different from children with normal vision, for whom luminance had no significant influence on the acuity threshold. Watson et al. [79] measured VEP Vernier acuity, VEP grating acuity, and behavioral PL acuity in patients with CVI, finding that VEP Vernier acuity was worse than grating acuity and more similar to PL acuity. Subsequently, Watson et al. [92] measured visual acuity with PL and VEP methods in young patients with CVI on two occasions, finding that early VEP acuity was almost the same as a future behavioral acuity measured about seven years later, even though the initial measurement results of VEP and PL varied greatly, demonstrating that sVEP testing can be utilized to predict future visual acuity in youngsters with CVI. Cavascan et al. [52] used sVEP to investigate the causal factors of acuity deficit and interocular acuity differences, finding variable severity of VEP acuity deficit in children with CVI. Pereira and Costa [111] measured the visual acuity of children with hydrocephalus with or without peritoneal–ventricular shunt by sVEP, suggesting that delayed insertion of the shunt may affect the visual development of these children.

Visual acuity testing was challenging in patients with cerebral palsy since their poor speech, gaze, and head control may affect testing [140,141]. Costa et al. [86] suggested that sVEP could provide a more precise and reliable estimate of visual acuity than behavioral measurements in children with spastic cerebral palsy since the behavioral acuity might be underestimated as a result of their motor impairment. They also found a strong relationship between sVEP visual acuity loss and quantified motor impairment. Tinelli et al. [130] tested sVEP and behavioral visual acuity in children with periventricular leukomalacia, showing a high correlation between sVEP and behavioral visual acuity. They suggested that behavioral measures could be a better expression of visual functionality since they reflected the effectiveness of the compensatory mechanisms after brain injury. Ghasia et al. [110] measured the visual acuity of children with cerebral palsy using optotype and SSVEP methods, concluding that they had a high likelihood of success, with 88% of children able to cooperate for either optotype or SSVEP assessment.

The VEP method was also used in the evaluation of visual acuity in amblyopia. Regan [142] used this technique by superposing a movie cartoon on the checkerboard pattern to assess visual acuity in young children with amblyopia, giving an index of acuity and acuity difference between the amblyopic and fellow eye. Odom et al. [127] used VEP as an acuity assessment method before and after eye patching, indicating no permanent deleterious effects of patching on acuity. Ridder and Rouse [94] compared sVEP acuity of pre-amblyopic therapy with Snellen acuity of post-amblyopic therapy, finding that sVEP acuity before the therapy can be used to predict Snellen acuity after therapy. Gundogan et al. [71] verified that pattern VEP can be used to predict objective visual acuity in amblyopic children by using the latency and amplitude of P100 in five consecutive check sizes. Wenner et al. [31] compared VEP acuity with psychophysical FrACT acuity in subjects with anisometropic and strabismic amblyopia, finding that acuity can be markedly overestimated using VEP in amblyopia even though measurements of both VEP and FrACT acuity were highly reproducible. Hou et al. [117] measured Vernier and grating acuity by sVEP and psychophysics to evaluate their validity and reliability of detecting amblyopia, suggesting that sVEP Vernier acuity provided a better index for the acuity defect of amblyopia than sVEP grating acuity.

Ddom et al. [32] verified the feasibility that VEP could be used to predict post-cataract visual acuity using a 10 Hz flash. Thompson et al. [103] utilized VEP as an objective tool to test visual acuity in children with congenital cataracts. Hanawa et al. [143] recorded preoperative VEP and found that VEP before cataract surgery was able to predict postoperative visual acuity in patients with glaucoma and cataract. Moreover, other studies also used VEP as an objective method to test visual acuity in patients with cataract and glaucoma [22,53,62,75,102,106,126,132,137,143].

In addition, Vadrevu et al. [33] tested SSVEP on diabetic eyes with vitreous hemorrhage using a 10 Hz flash and assessed the utility of SSVEP in predicting the final visual acuity of these patients. Faria et al. [82] evaluated visual abnormalities without retinopathy in patients with type 1 diabetes mellitus by the VEP method and found that those patients demonstrated significant lower VEP amplitude at all spatial frequencies compared with normal subjects, indicating an optic nerve dysfunction in patients with type 1 diabetes mellitus. John et al. [88] tested visual acuity by VEP and behavioral methods, finding that visual acuity was lower in children with Down syndrome than those not suffered. They also suggested the idea of an underlying sensory deficit in the visual system in Down syndrome. Bradfield et al. [123] suggested that sVEP testing can be recognized as a predictor of visual acuity in children with albinism after testing sVEP at specific intervals (6, 12, 18, and 24 months). McBain et al. [107] used VEP as a clinical tool in the objective estimation of patients with suspected non-organic visual loss. Hamilton et al. [105] used VEP as a method of estimating acuity in children with reduced visual acuity and clinical suspicion of functional visual loss, suggesting high specificity of the VEP test in diagnosing functional visual loss.

Some studies measured VEP acuity in patients with various visual disorders, as listed in Table 3, and we classified these visual disorders into different categories (e.g., nystagmus, macular diseases, retinal diseases, optic nerve disorders, structural anomalies, eye trauma, and delayed visual maturation trauma). Sokol et al. [106] measured VEP and behavioral FPL acuity in infants and children with normal vision, amblyopia, and other various disorders (e.g., cataract, vitreous hemorrhage, retrolental fibroplasia, congenital nystagmus, etc.), finding that children younger than 2 years old could be more successfully tested by VEP than by FPL, and VEP acuity was more consistent with clinically determined acuity than FPL acuity. Steele et al. [102] assessed the clinical utility of visual acuity measurement by VEP in emmetropic subjects, medical subjects with uncorrected myopia, patients with known ocular disease (e.g., glaucoma, background diabetic retinopathy, vitreous hemorrhage, retinitis pigmentosa, cataract, toxic optic neuropathy, etc.), and patients with suspected functional visual loss, indicating that VEP can be a valid method to assess visual acuity in healthy subjects with refractive errors and patients with different ocular diseases, and can also objectively diagnose functional visual loss. Gottlob et al. [75] assessed sVEP and optotype acuity in children with various visual disorders (e.g., amblyopia, aphakia, cataract, nystagmus, albinism, retinopathy of prematurity, retinal colobomas, etc.), finding a high correlation coefficient between VEP and optotype acuity (r = 0.6–0.89), suggesting that VEP can be a valid method of estimating visual acuity in the clinical management of non-verbal patients.

Later, Gottlob et al. [54] compared sVEP and recognition acuity in youngsters with organic diseases, nystagmus, strabismus without amblyopia, and congenital ptosis without amblyopia. They found a high correlation (r = 0.97) between sVEP and recognition acuity in patients with organic diseases, and youngsters with alternating fixation and strabismus (r = 0.92), but a nonsignificant correlation (r = 0.61, *p* > 0.05) in nystagmus. Bobak et al. [70] measured visual acuity by the VEP method in patients with ambiguous acuity loss (e.g., trauma, enucleation, glaucoma, strabismic amblyopia, etc.), suggesting that VEP can be useful as a measurement of pathway integrity. Katsumi et al. [62] found a good relationship (r = 0.847) between PL and VEP acuity in youngsters with various ocular pathologies (e.g., amblyopia, congenital cataract, cone dysfunction syndrome, glaucoma, congenital retinoschisis, persistent hyperplastic primary vitreous, etc.) except a dissociation in those with very low vision. Arai et al. [63] additionally compared VEP and Snellen acuity in patients with different ocular pathologies (e.g., macular diseases, diffuse retinal degeneration, optic nerve diseases, glaucoma, high myopia, etc.), finding a high correlation, but a low correlation in patients with optic nerve disease. Westall et al. [67] found an inconsistency between VEP acuity and TAC source in children with various ocular and neurologic conditions (e.g., macular abnormality, retinal abnormality, optic nerve hypoplasia, optic nerve atrophy, cortical visual impairment, developmental delay, cerebral palsy, seizures, nystagmus, etc.), suggesting a consistent method for visual acuity assessment of children during visual development. Sobaci et al. [34] verified a 10 Hz flash VEP to predict postoperative visual acuity in severe eye trauma with opaque media. Rao et al. [132] explored the accuracy of VEP acuity estimation in 726 patients with a post-traumatic unilateral decrease in visual acuity (e.g., eyelid contusion, hyphema, traumatic lens lesion, vitreous hematocele, retinal lesion, optic nerve contusion, etc.), but obtained only a coincidence rate of 17.5% between subjective and VEP acuity. Jeon et al. [112] validated the use of VEP to measure visual acuity in people with normal vision and patients with unilateral amblyopia, optic neuritis, and visual disability, suggesting a cutoff value of 5.77 µV for discriminating malingering from real disability.

In summary, one of the most essential clinical applications of VEP acuity assessment is in pediatric testing, i.e., in preverbal children or children with cognitive impairments, to measure visual acuity objectively. If VEP acuity is within a normal range corresponding to age, it can be deduced that the function of the visual pathway from the eyeball to the cortex is intact. Moreover, VEP is identified as a valuable and alternative approach to measure visual acuity in visual disorders. In general, behavioral and VEP acuity are relatively close in patients with media opacity, refractive errors, and retinal dysfunction. However, VEP acuity has poor accuracy and precision compared to behavioral acuity when visual disorders are due to abnormality of the macula, the optic nerve, or any cerebral structures.

## 6. Discussion and Conclusions

VEP has been used as an alternative method to assess visual acuity objectively, especially in non-verbal infants and adults with low intellectual abilities or malingering. This review summarizes the technique of VEP acuity assessment from some aspects including the parameter settings of visual stimuli, signal acquisition and analysis methods, and clinical application. However, VEP acuity assessment has not been an extensively accepted objective method due to the lack of a standardized protocol. Researchers and clinicians need to set a common standard for the VEP acuity test technique since so many parameter settings must be considered during test system construction, experimental testing, and data processing and analysis. A common standard would enable the technique to be used more widely and quickly, deepening VEP and even electrophysiology research on the detection and diagnosis of visual function. In this way, the increasingly severe global situation of myopia and vision impairment can be somewhat relieved by this early visual acuity evaluation method.

Another challenge is that the agreement between VEP and behavioral acuity is not always good, which may be caused by the difference of the assessing entity between VEP and behavioral tests. Here we offer some possible reasons. First, compared to behavioral acuity, with decisions made quickly, VEP needs a longer time due to the requirement of sufficient data [144]. Next, the higher cognitive cortex is involved in the behavioral test, but VEP is only cellular activity in the primary visual cortex [145]. Finally, compared to the stationary targets of behavioral acuity, VEP acuity utilizes dynamic targets. Hence, VEP acuity is not always close to behavioral acuity in some groups, such as patients with optic nerve disease, macular disease, or amblyopia.

VEP can be a complementary tool for visual acuity estimation and even an indispensable measure when a behavioral test is not suitable, such as in pediatric testing. In general, VEP acuity increases from 1 to 20 cpd during the first year of life, and then reaches adult levels between 2 and 10 years old. It is necessary to establish age norms of VEP acuity in infants and children, since behavioral VEP, e.g., PL acuity, is not objective enough for little children. Hence, VEP acuity at an early age can be an index to assess the integrity of the early visual pathways from the optics to the cortex.

With the development of technology, VEP acuity has broad application prospects. First, increasing computing power can minimize the test duration and improve test efficiency. Second, the widely used signal analysis method in the VEP-based brain-computer interface, e.g., canonical correlation analysis [7] and multivariate synchronization index [146], can be used to promote the performance of VEP acuity assessment. Third, the eye-tracking technique can record the eyeball position, which can automatically intercept EEG epochs when the examinee stares at visual stimuli, which is especially relevant for infants [147]. Finally, artificial intelligence technology, e.g., machine learning and deep learning, may offer an alternative method to establish a mathematical model of VEP response and acuity other than the threshold definition [27].

## Figures and Tables

**Table 2:** Studies of visual acuity development using VEP.

First Author	Year	Subjects’ Age Range	Results
Sokol [100]	1978	Infants: 2–6 months Adults	VEP acuity improved from 20/150 at 2 months to 20/20 by 6 months.
Norcia [13]	1985	Infants: 17–25 weeks	Temporal frequency of 6 or 10 Hz did not affect estimation of sVEP acuity. Sweep technique was also a robust method for measuring visual acuity.
Norcia [14]	1985	Infants: 1–53 weeks Adults	VEP acuity increased from 4.5 cpd in the first month to about 20 cpd at 8–13 months. By 8 months, VEP acuity reached adult level.
Sokol [73]	1988	Infants: 2–10 months Adults	Grating acuity was temporally tuned at 7.5 or 14 rps for infants at 3 months and older. Difference between VEP and PL acuity decreased from 2.0 octaves at 2 months to 0.5 octaves at 12 months.
Hamer [124]	1989	Infants: 2–52 weeks	Monocular and binocular acuity growth functions were nearly identical; monocular and binocular VEP acuity increased from 6 cpd at 2–10 weeks to 14 cpd by 20–30 weeks.
Norcia [65]	1990	Infants: 2–40 weeks Adults	SVEP estimated grating acuity showed gradual increase with age, ranging from 2.5–9 cpd in the first 2 months to about 10–20 cpd after 30 weeks.
Sokol [74]	1992	Infants: 2–13 months Children: 1–5 years Adults: 22–48 years	VEP and PL acuity developed at different rates, reaching a nearly equivalent level by 12 months. PL acuity in infants older than 2 years was found to be not temporally tuned.
Allen [19]	1992	Infants: 15–20 weeks Adults	FPL acuity improved slightly more with luminance than did VEP acuity. Acuity levels of VEP and FPL were comparable, with VEP slightly higher.
Riddell [121]	1997	Pre-term infants: 2–8 months Full-term infants: 3 weeks to 1 year	VEP acuity was generally higher than TAC acuity, but the rate of development was higher for TAC than VEP. TAC acuity reached VEP acuity at about 14 months. There was no difference between pre-term and full-term infants in VEP and TAC acuity.
Skoczenski [66]	1999	Infants: 8–80 weeks Adults	VEP Vernier and grating acuity developed at different rates, the former approaching adult levels earlier than the latter. Vernier acuity increased by a factor of 4.5 between 10 and 100 weeks; grating acuity improved by a factor of 2.3.
Prager [93]	1999	Infants: 4–8 months	Correlations among transient VEP, sVEP, and TAC acuity were poor, but expected changes in visual maturation from 4 to 8 months were detected with all methods. SVEP acuity increased from 9.61 cpd at 4 months to 10.39 cpd at 8 months.
Suttle [91]	2000	Infants: 6–17 weeks	Most infants did not exhibit clear VEP to whole-field flicker alone. Estimated VEP acuity was generally not confounded by front-end nonlinear distortion products.
Maria [101]	2001	Infants: 15.2–17.7 weeks	Perinatal characteristics including birth weight, gender, and number of smokers in the household needed to be considered for VEP acuity.
Lauritzen [81]	2004	Infants: 6–40 weeks	Mean rather than maximum threshold best estimated visual acuity. VEP method was well suited to describe visual development in infants, which increased by 0.64 octaves per doubling in age for acuity.
Salomão [99]	2008	Infants/children: 1–36 months	Age norms for grating acuity were determined using sweep VEP technique. Sweep VEP grating acuity ranged from 0.80 logMAR in the first month to 0.06 logMAR at 36 months.
Lenassi [35]	2008	Infants/children: 1.5 months to 7.5 years	VEP latency was strongly associated with visual acuity, recommending VEP latency as a reliable parameter for evaluating the integrity of the afferent visual pathway.
Almoqbel [109]	2017	Children: 6–7, 8–9, 10–12 years Adults	Results of various procedures (sweep VEP, psychophysical logMAR letter, and grating visual acuity) were in agreement. There were age-related changes in the visual acuity threshold after 6 years of age and visual acuity did not become adult-like until 8 to 9 years at the earliest.

**Table 3:** Clinical studies of VEP acuity for visual disorders.

Categorization	Detailed Disorder Types	Studies
Cortical visual impairment	Hypoxic injury, infection, hydrocephalus	[20,52,67,75,79,90,92,111]
Cerebral palsy	Tetraplegic, diplegic, hemiplegic, periventricular leukomalacia	[67,86,110,129,130]
Amblyopia	Refractive amblyopia, strabismic amblyopia, deprivation	[31,54,62,63,70,71,75,94,106,112,117,123,126,127,142]
Cataract		[22,32,53,62,75,102,103,106,126,137,143]
Glaucoma		[53,62,63,70,75,102,143]
Albinism		[54,75,80,123]
Diabetes	Type 1 diabetes mellitus, background diabetic retinopathy, diabetes with vitreous hemorrhage	[33,80,82,102]
Down syndrome		[88]
Functional visual loss		[102,105,107,112]
Nystagmus	Congenital nystagmus, infantile nystagmus, spasmus nutans	[53,54,67,75,106,123]
Macular diseases	Macular gliosis, macular holes, macular degeneration, age-related macular degeneration, cystoid macular edema, maculopathy, neurosensory macular detachment, macular abnormality, foveal hypoplasia, retinal pigment epithelium macular detachment, Stargardt’s disease, central serous retinopathy	[22,53,63,67,80,102,123,137]
Retinal diseases	Retinitis pigmentosa, retinal reattachment, congenital retinoschisis, cone dysfunction syndrome, congenital retinoschisis, retina coloboma, retinopathy of prematurity, diabetic retinopathy, retinal myelin, rod/cone dystrophy, lattice degeneration, peripheral retinal holes, juvenile X-linked retinoschisis, retinal detachment, retinal perforation, epiretinal gliosis, chorioretinitis	[12,22,54,62,63,67,80,102,106,132,137]
Optic nerve disorders	Optic neuritis, optic atrophy, optic nerve hypoplasia, optic glioma, Leber’s atrophy, toxic optic neuropathy, cortical blindness, 3rd nerve palsies	[54,63,70,75,84,102,106,112,129,137]
Structural anomalies	High myopia, persistent hyperplastic primary vitreous, vitreous hemorrhage, refractive error, ptosis, iris and choroid coloboma, persistent hyperplastic primary vitreous, retrolental fibroplasia, vitreous hemorrhage, sub-hyaloid hemorrhage, vitreous opacity, aphakia, microphthalmia, corneal clouding	[22,53,54,62,63,75,102,106,123,126,137]
Eye trauma	Severe eye trauma with opaque media, eyelid contusion, hyphema, traumatic lens lesion, vitreous hematocele, retinal lesion, optic nerve contusion	[34,70,79,80,132]
Delayed visual maturation		[35,75]
